# Supplementary material for: Low pressure reversibly driving colossal barocaloric effect in two-dimensional vdW alkylammonium halides
Source: Nat Commun. 2024 Feb 28;15:1838. doi: 10.1038/s41467-024-46248-1 (PMC10901796; doi:10.1038/s41467-024-46248-1)
Supplement: Supplementary file 1 — Supplementary Information [file 41467_2024_46248_MOESM1_ESM.pdf]

Supplementary Information

Low pressure reversibly driving colossal barocaloric effect in two-dimensional vdW alkylammonium halides

Yi-Hong Gao[a,b#], Dong-Hui Wang[c#], Feng-Xia Hu[a,b,d*], Qing-Zhen Huang[e,f], You-Ting Song[a,b], Shuai-Kang Yuan[a,b], Zheng-Ying Tian[a,b], Bing-Jie Wang[a,b], Zi-Bing Yu[a,b], Hou-Bo Zhou[a,b], Yue Kan[a,b], Yuan Lin[a,b], Jing Wang[a,b*], Yun-liang Li[a,b,d*], Ying Liu[c], Yun-Zhong Chen[a,b], Ji-Rong Sun[a,b,d], Tong-Yun Zhao[a,g], Bao-Gen Shen[a,b,e*]

[a]Beijing National Laboratory for Condensed Matter Physics, Institute of Physics, Chinese Academy of Sciences, Beijing, 100190, P. R. China.

[b]School of Physical Sciences, University of Chinese Academy of Sciences, Beijing, 101408, P. R. China.

[c]College of Chemistry, Beijing Normal University, 100875 Beijing, P. R. China

[d]Songshan Lake Materials Laboratory, Dongguan, Guangdong, 523808, P. R. China.

[e]Ningbo Institute of Materials Technology & Engineering, Chinese Academy of Sciences, Ningbo, Zhejiang, 315201, China.

[f]Spallation Neutron Source Science Center, Dongguan, 523803, China

[g]Ganjiang Innovation Academy, Chinese Academy of Sciences, Ganzhou, Jiangxi, 341000, China.

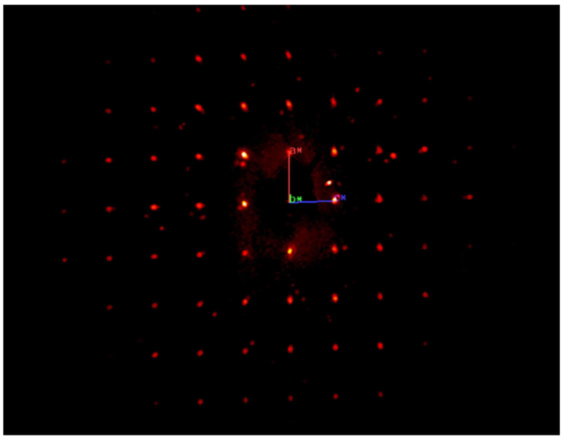

Supplementary Fig. S1 The diffraction point patterns of $dC_{10}Cl$ at 300 K for the low-temperature-state.

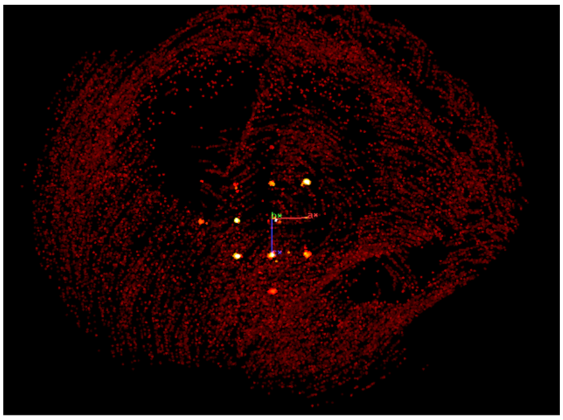

Supplementary Fig. S2 The diffraction point patterns of $dC_{10}Cl$ at 340 K for the high-temperature-state.

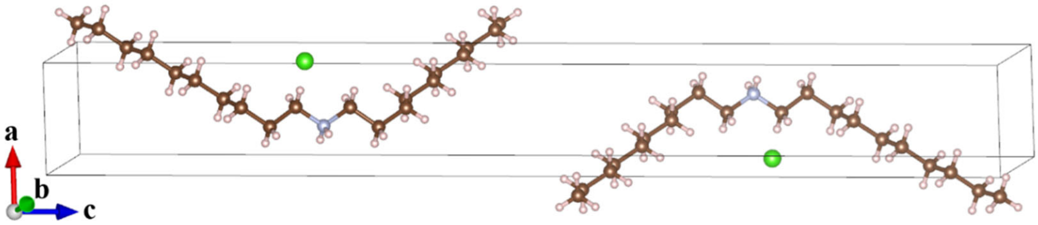

Supplementary Fig. S3 The molecular structure in the unit cell of $dC_{10}Cl$ at 300 K refined from SC-XRD result.

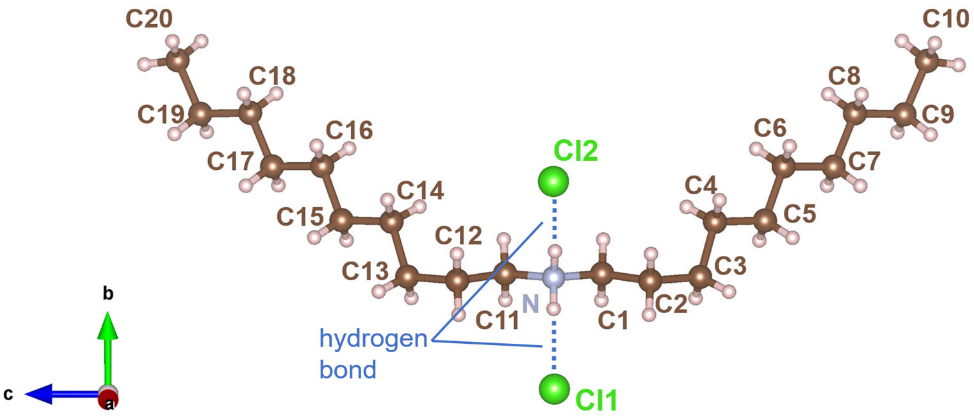

Supplementary Fig. S4 The structure and hydrogen bond environment of alkyl chains $((CH_3–(CH_2)_9)_2NH_2)^+$ in $dC_{10}Cl$.

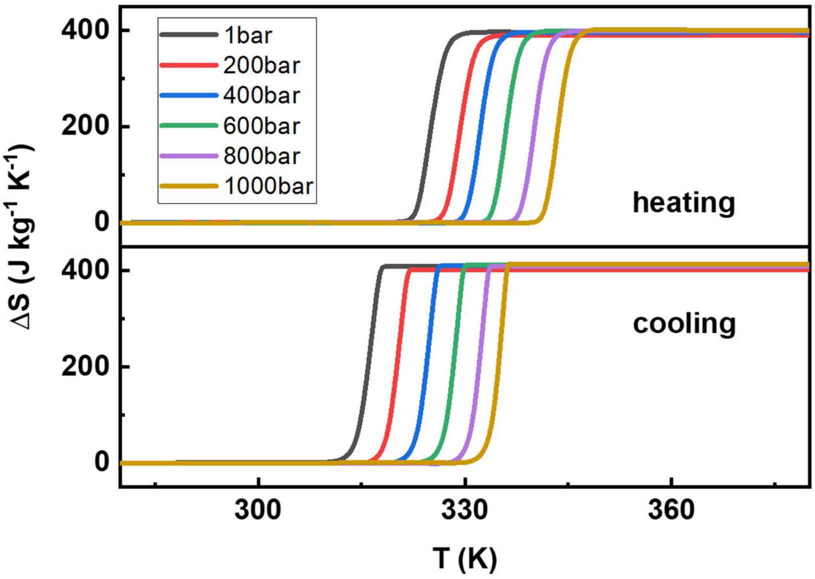

Supplementary Fig. S5 The entropy change curves of only phase transition under variable pressures from the integral of heat flow curves in $dC_{10}Cl$.

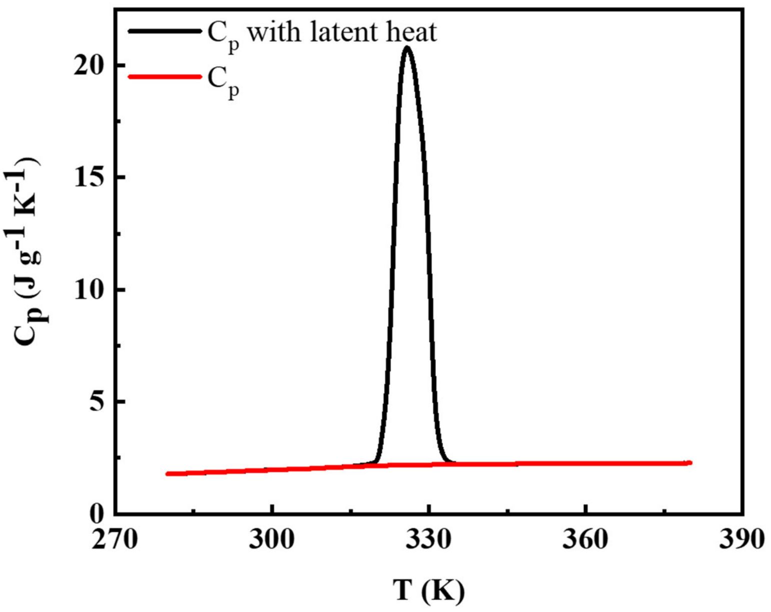

Supplementary Fig. S6 The specific heat capacity of $dC_{10}Cl$ measured under ambient pressure.

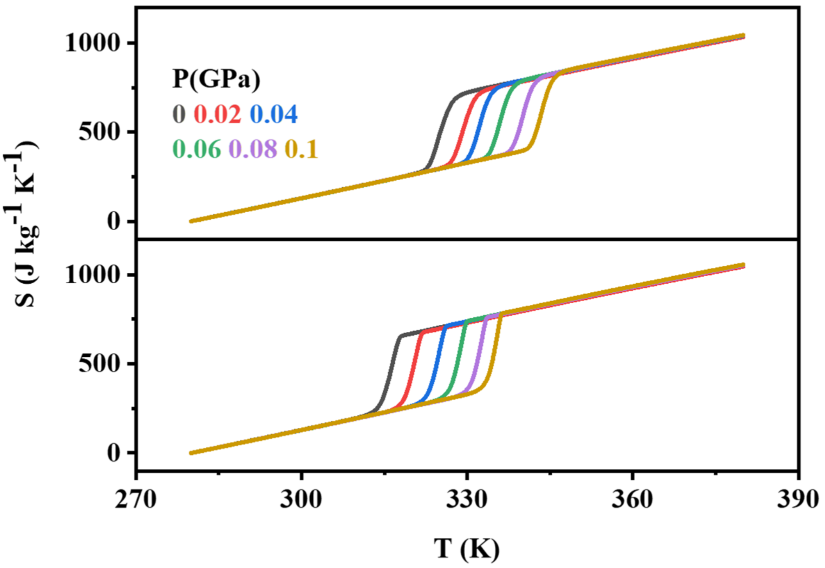

Supplementary Fig. S7 Total entropy containing both specific heat and phase transition under variable pressures.

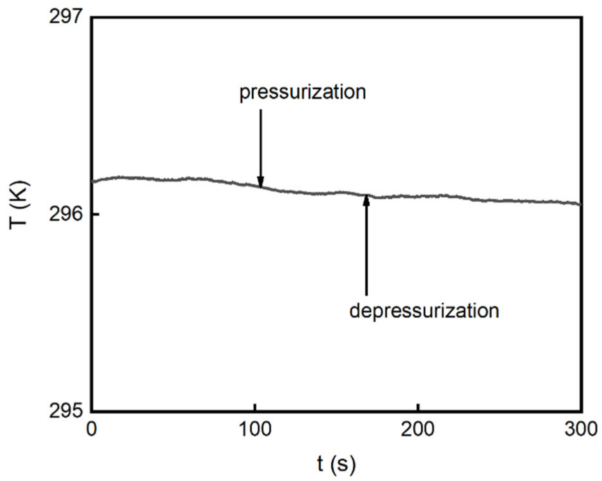

Supplementary Fig. S8 Direct measurement of adiabatic temperature change on NaCl upon pressurization and depressurization, where the pressure ranges from 0 to 0.1 GPa. To make the measured adiabatic temperature change (ΔT_{ad}) reliable, we chose NaCl as a benchmark and performed the direct measurement of ΔT_{ad} before each measurement for present $dC_{10}Cl$. A typical result is given in Fig. S8. At ~296 K, a pressure of 0.1 GPa was applied on NaCl and then released. The temperature error is within 0.1 K. One can see there are no temperature change detected during either pressurization or depressurization process, which illustrated the well-calibrated ΔT_{ad} for the present $dC_{10}Cl$.

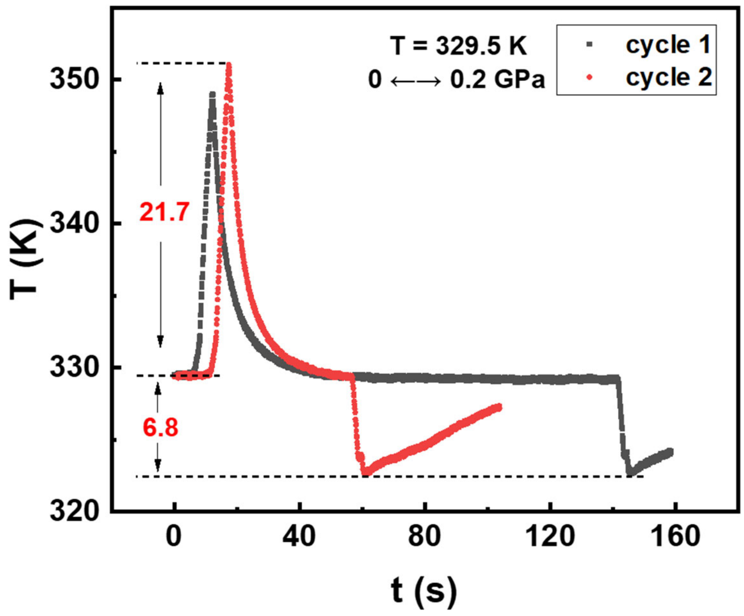

Supplementary Fig. S9 Cyclic measurements of adiabatic temperature change on $dC_{10}Cl$ in the pressure range of 0–0.2 GPa.

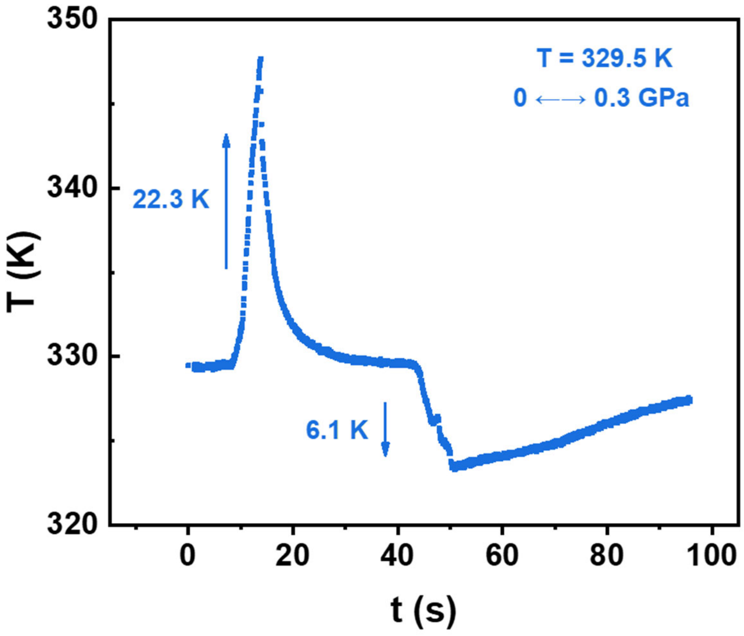

Supplementary Fig. S10 Adiabatic temperature change measurements on $dC_{10}Cl$ in the pressure range of 0–0.3 GPa.

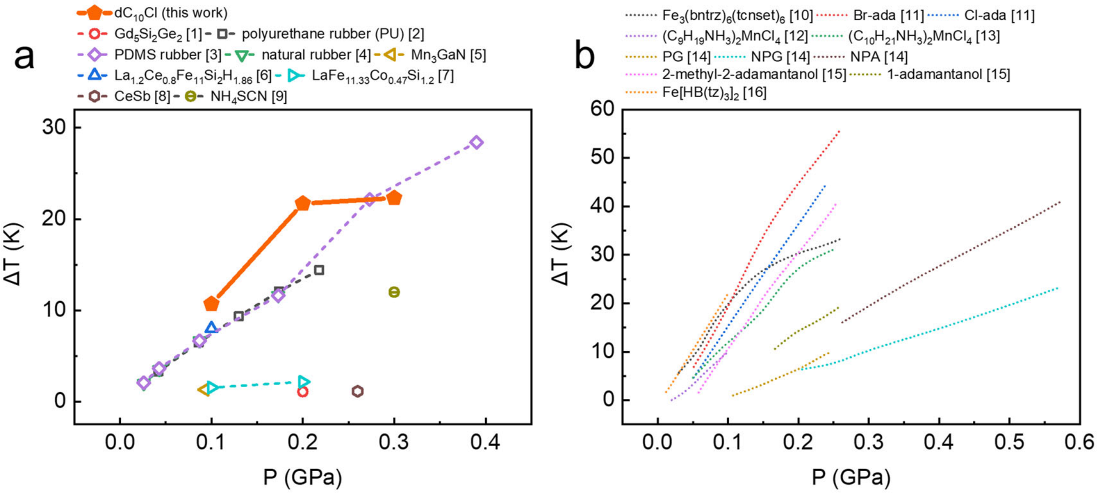

Supplementary Fig. S11 (a) The directly measured reversible adiabatic temperature change ΔT of our $dC_{10}Cl$ compared to other barocaloric materials we can find in the literatures[1-9]. (b) The reversible ΔT of some representative colossal barocaloric materials with phase transition entropy change near or above 100 J kg^{-1} K^{-1} by quasi-direct method[10-16].

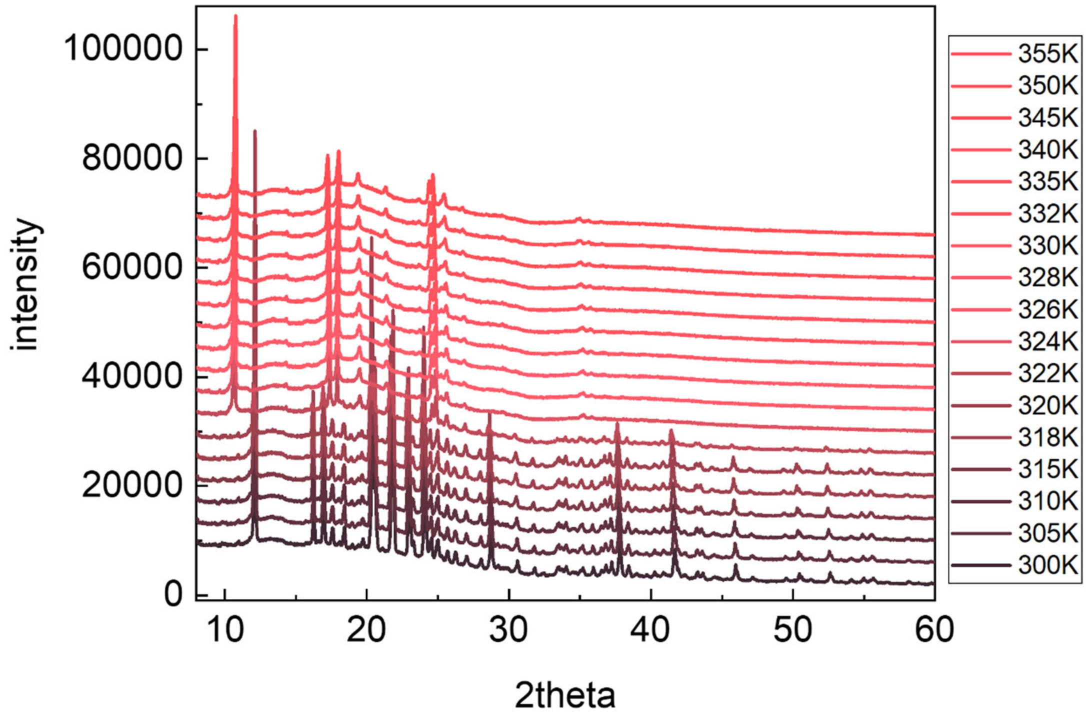

Supplementary Fig. S12 Temperature-variable powder x-ray diffraction patterns of $dC_{10}Cl$ on heating in the range of 300–350 K.

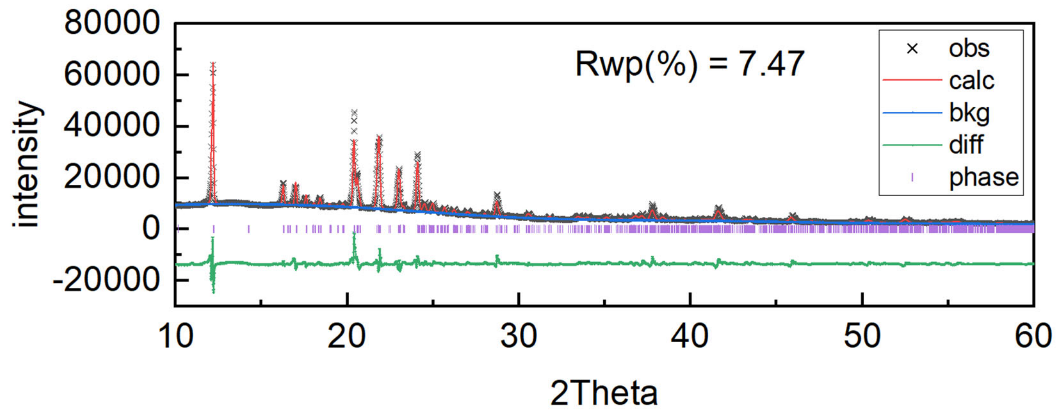

Supplementary Fig. S13 Rietveld refined pattern of $dC_{10}Cl$ at 305 K. The observed (black), calculated patterns (red), their difference (green), peak positions (purple bar), background (blue), and error factor Rwp are provided.

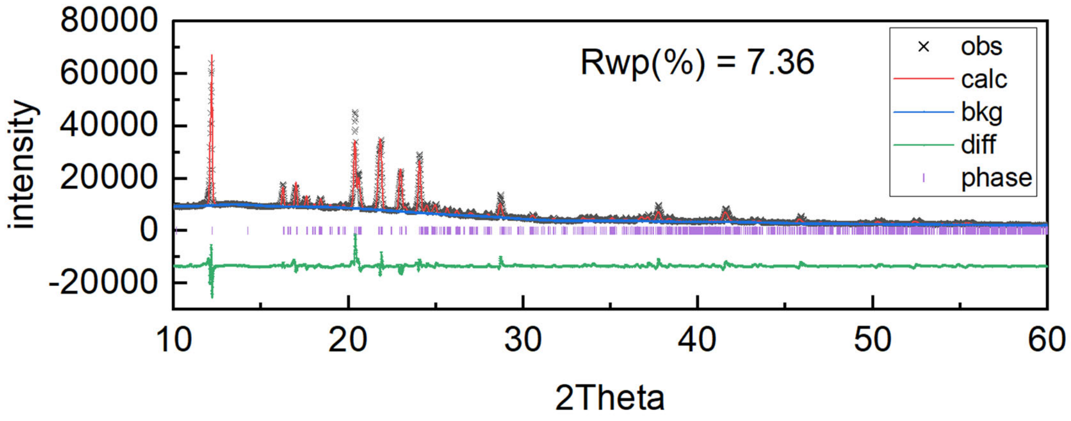

Supplementary Fig. S14 Rietveld refined pattern of $dC_{10}Cl$ at 310 K.

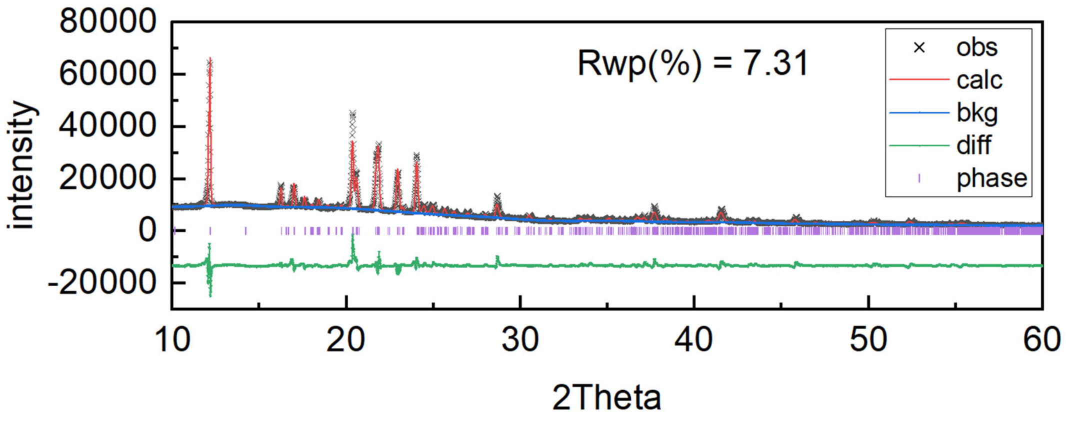

Supplementary Fig. S15 Rietveld refined pattern of $dC_{10}Cl$ at 315 K.

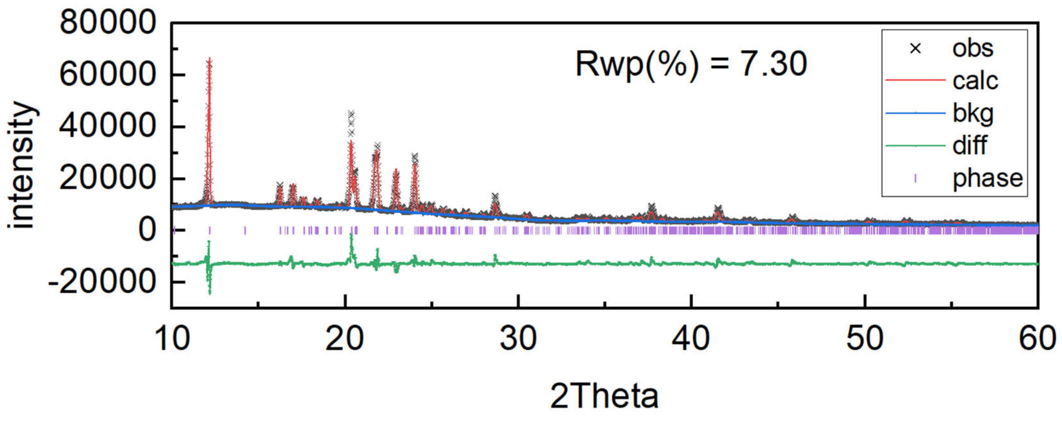

Supplementary Fig. S16 Rietveld refined pattern of $dC_{10}Cl$ at 318 K.

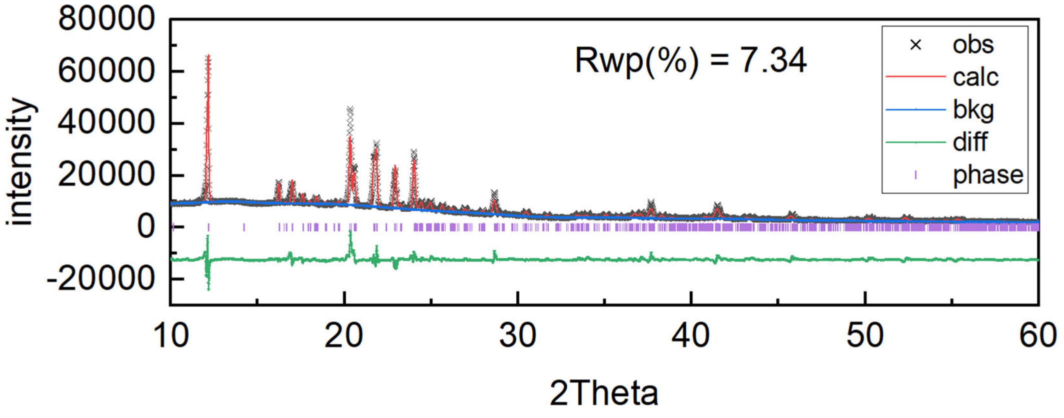

Supplementary Fig. S17 Rietveld refined pattern of $dC_{10}Cl$ at 320 K.

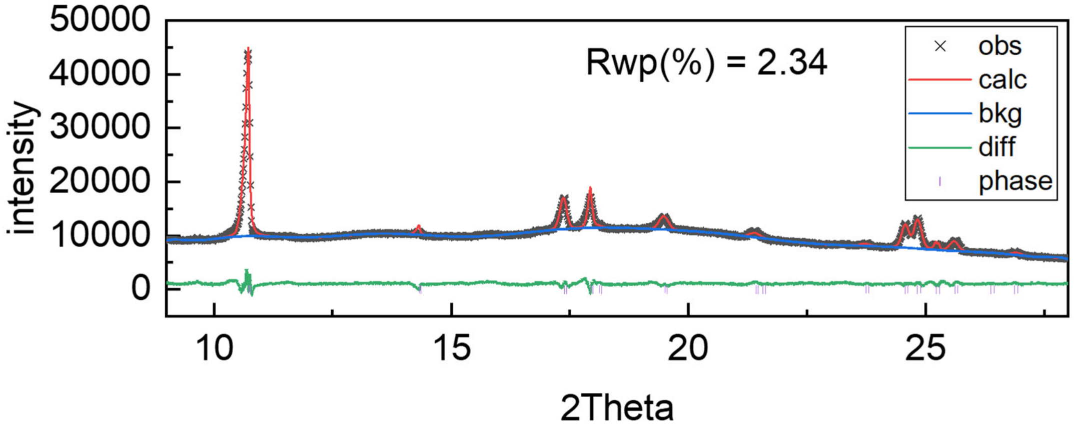

Supplementary Fig. S18 Le bail refined pattern of $dC_{10}Cl$ at 324 K. The observed (black), calculated patterns (red), their difference (green), peak positions (grey bar), background (blue), and error factor Rwp are provided.

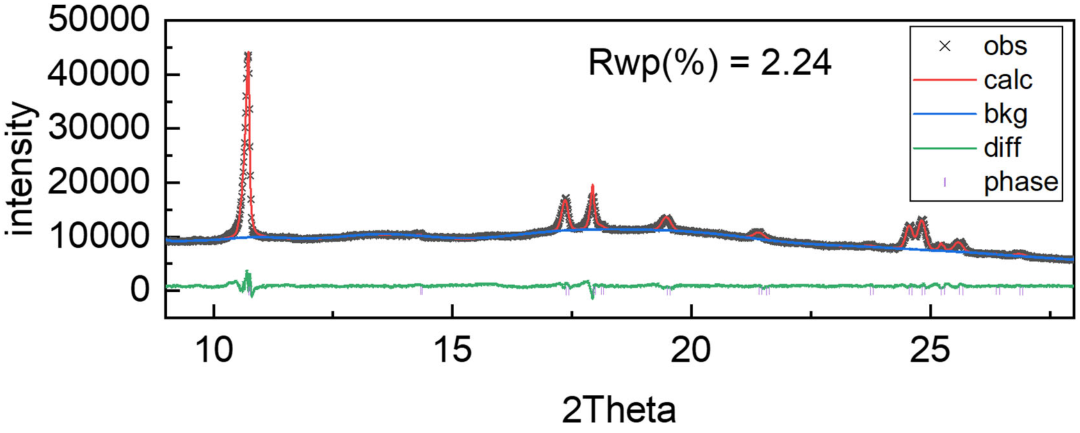

Supplementary Fig. S19 Le bail refined pattern of $dC_{10}Cl$ at 326 K.

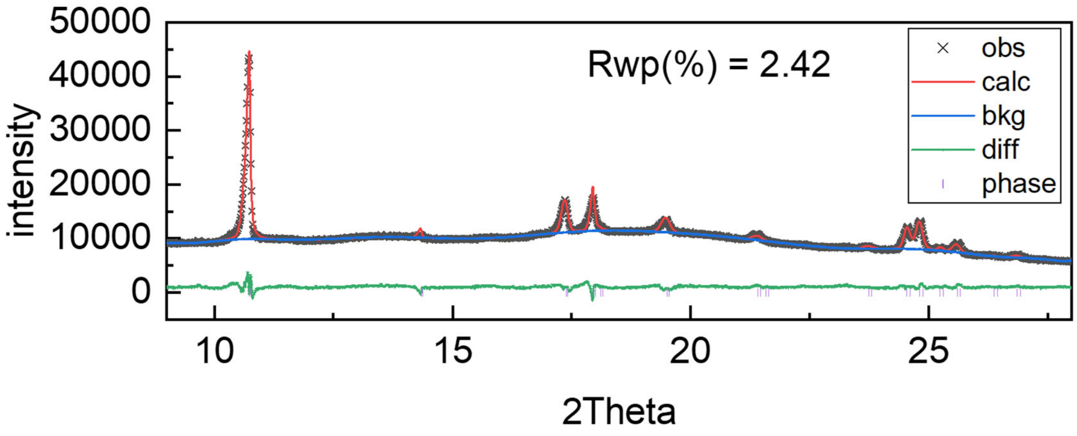

Supplementary Fig. S20 Le bail refined pattern of $dC_{10}Cl$ at 328 K.

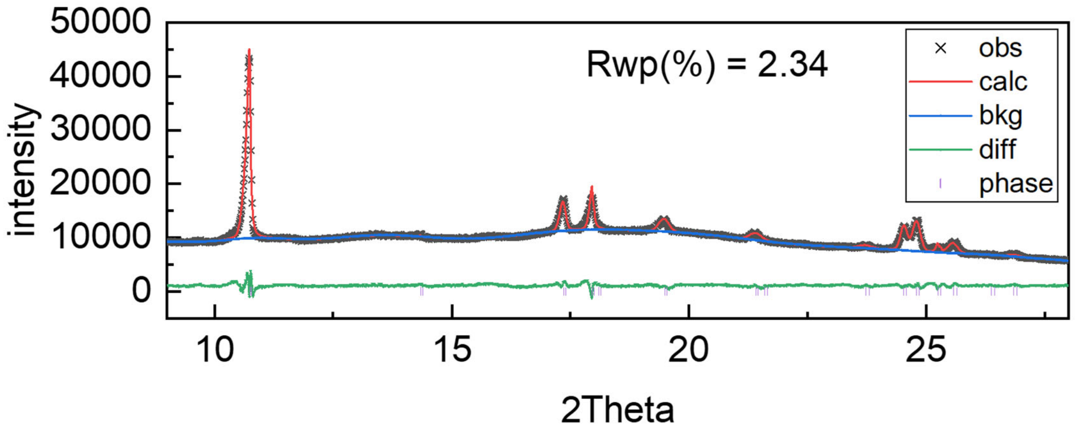

Supplementary Fig. S21 Le bail refined pattern of $dC_{10}Cl$ at 332 K.

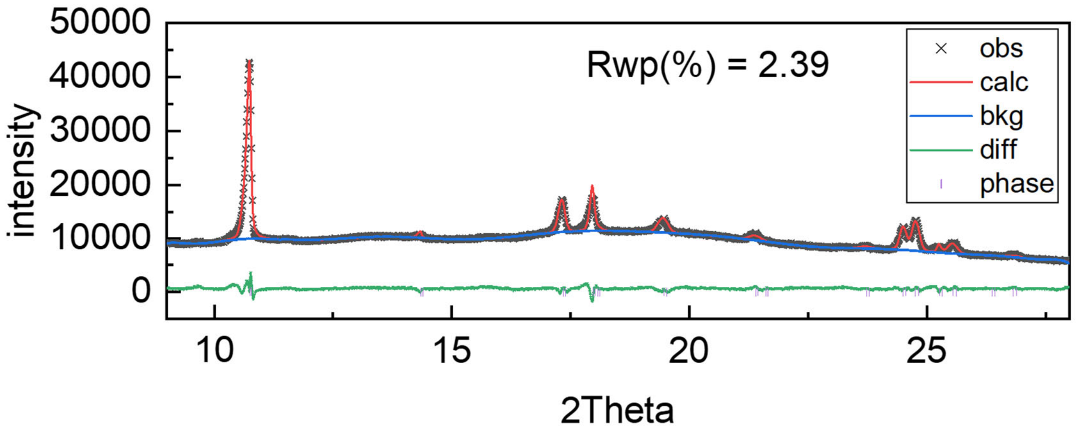

Supplementary Fig. S22 Le bail refined pattern of $dC_{10}Cl$ at 335 K.

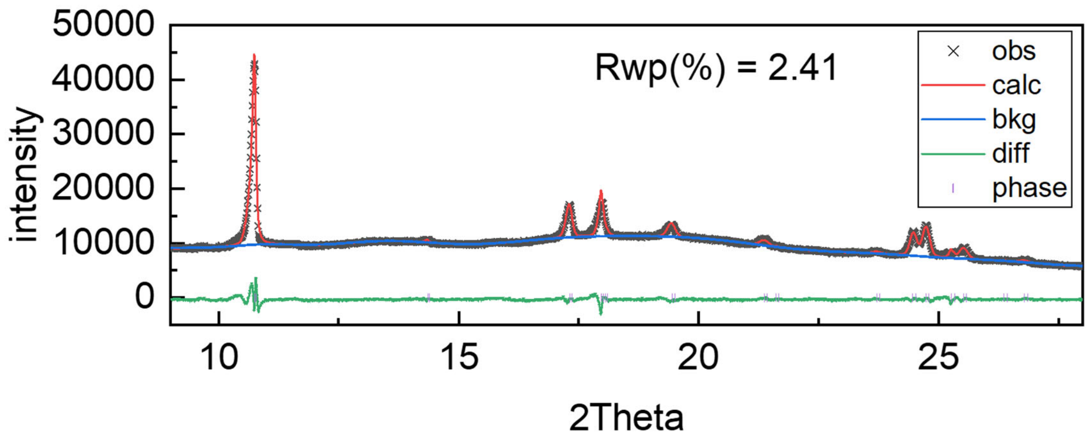

Supplementary Fig. S23 Le bail refined pattern of $dC_{10}Cl$ at 340 K.

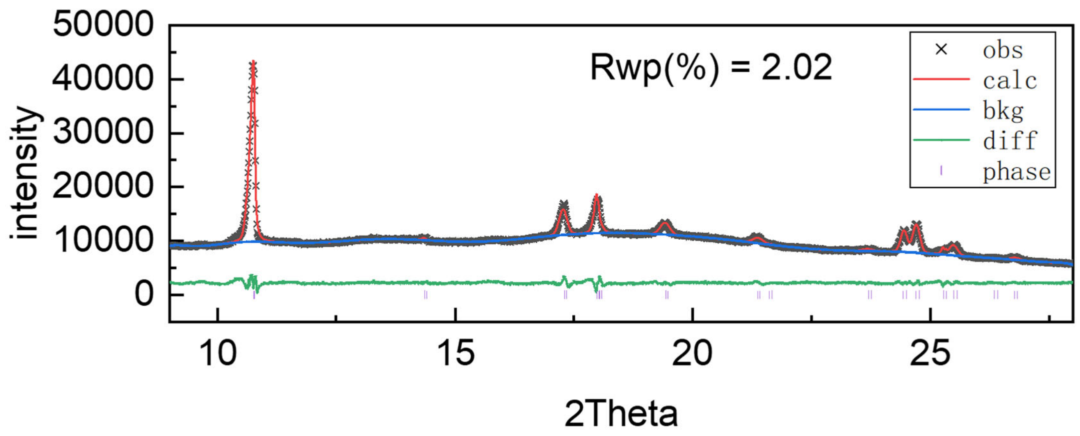

Supplementary Fig. S24 Le bail refined pattern of $dC_{10}Cl$ at 345 K.

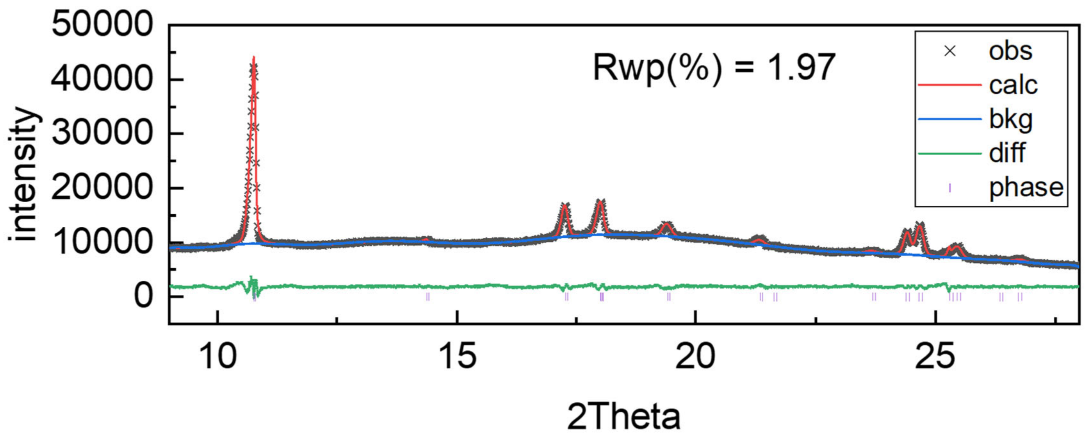

Supplementary Fig. S25 Le bail refined pattern of $dC_{10}Cl$ at 350 K.

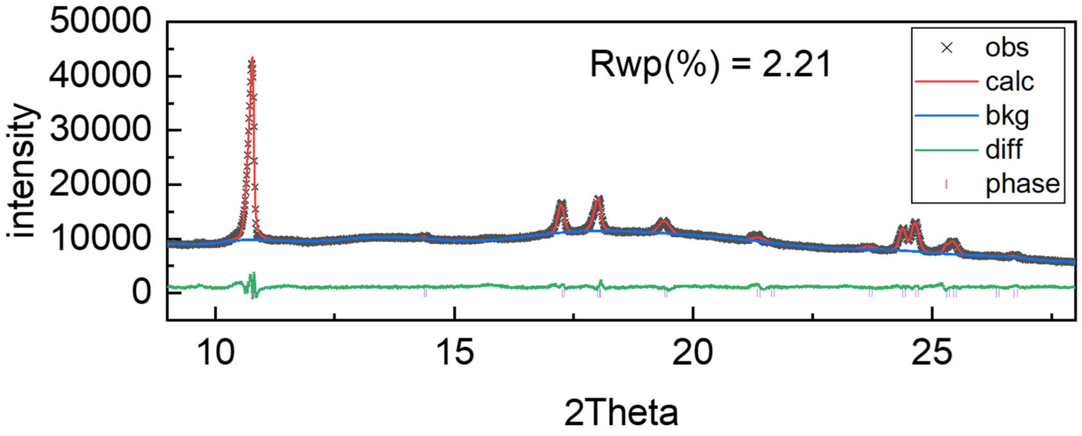

Supplementary Fig. S26 Le bail refined pattern of $dC_{10}Cl$ at 355 K.

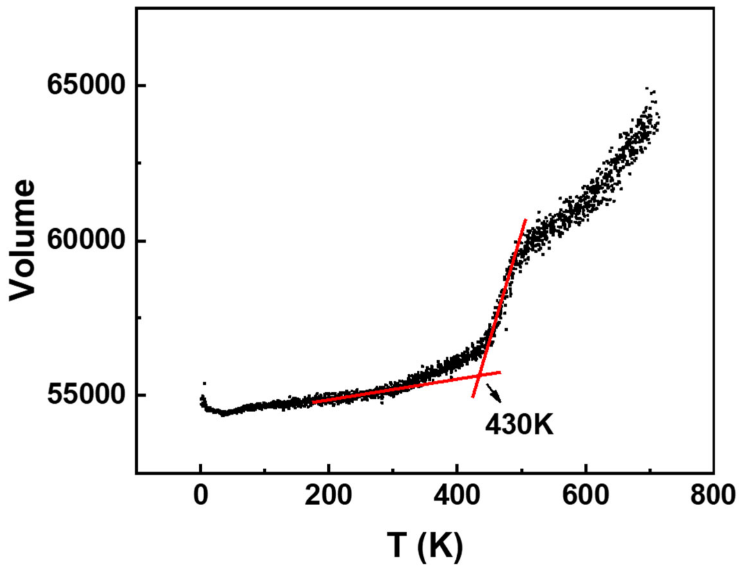

Supplementary Fig. S27 The volume evolution with respect to temperature from molecular dynamics (MD) simulation.

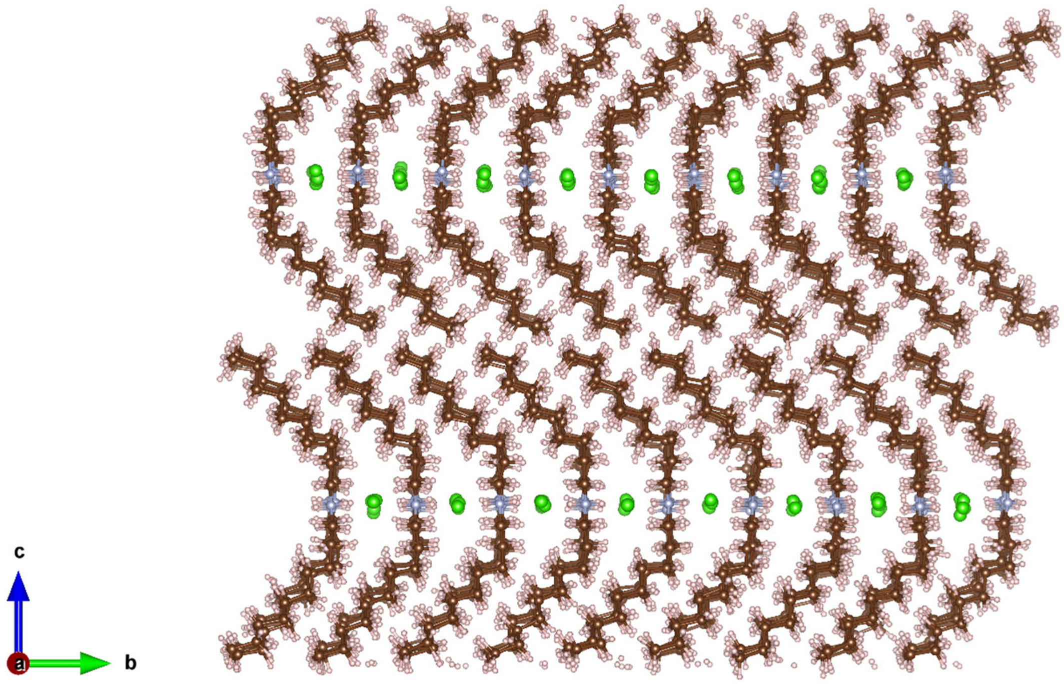

Supplementary Fig. S28 The crystalline structure of $dC_{10}Cl$ at 300 K evolved from the MD simulation.

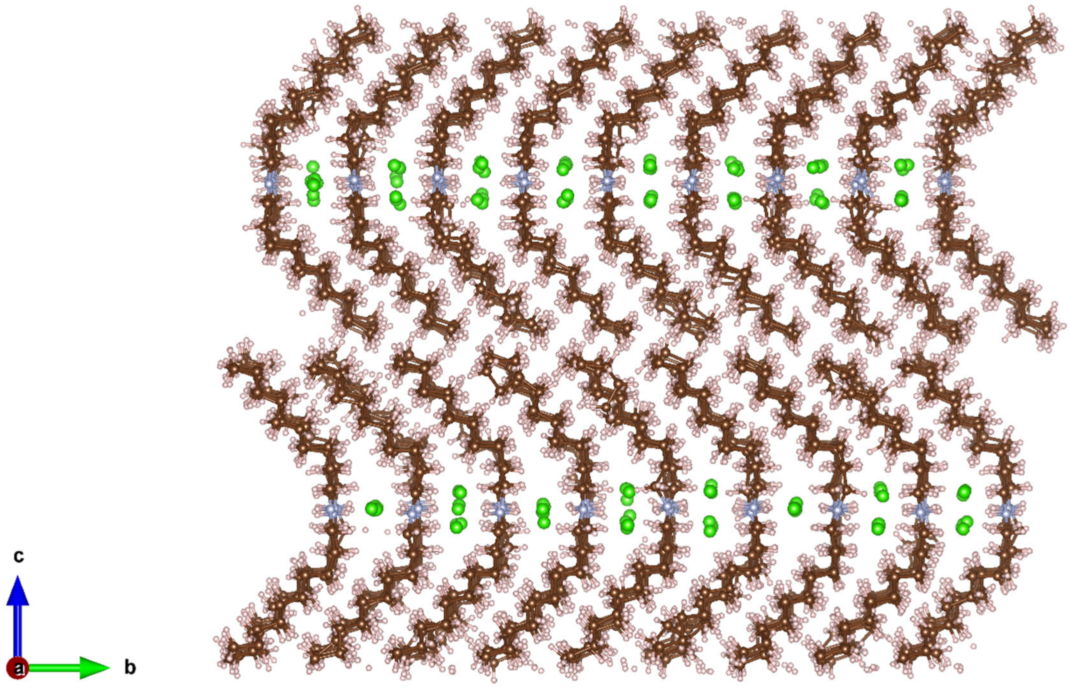

Supplementary Fig. S29 The crystalline structure of $dC_{10}Cl$ at 400 K evolved from the MD simulation.

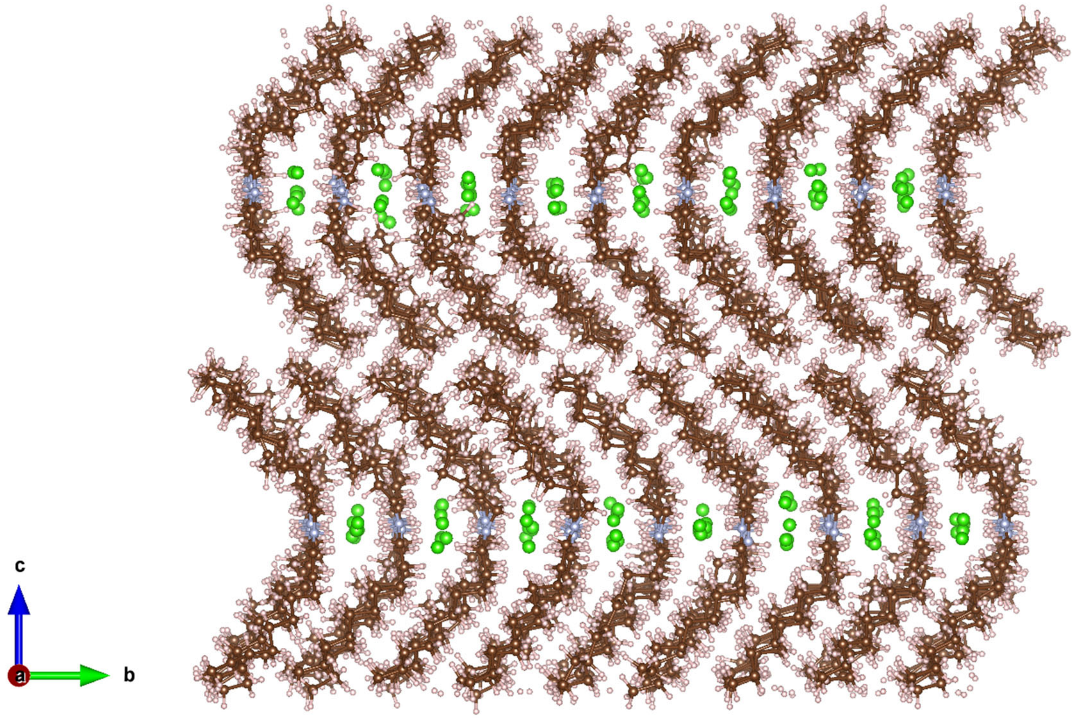

Supplementary Fig. S30 The crystalline structure of $dC_{10}Cl$ at 450 K evolved from the MD simulation.

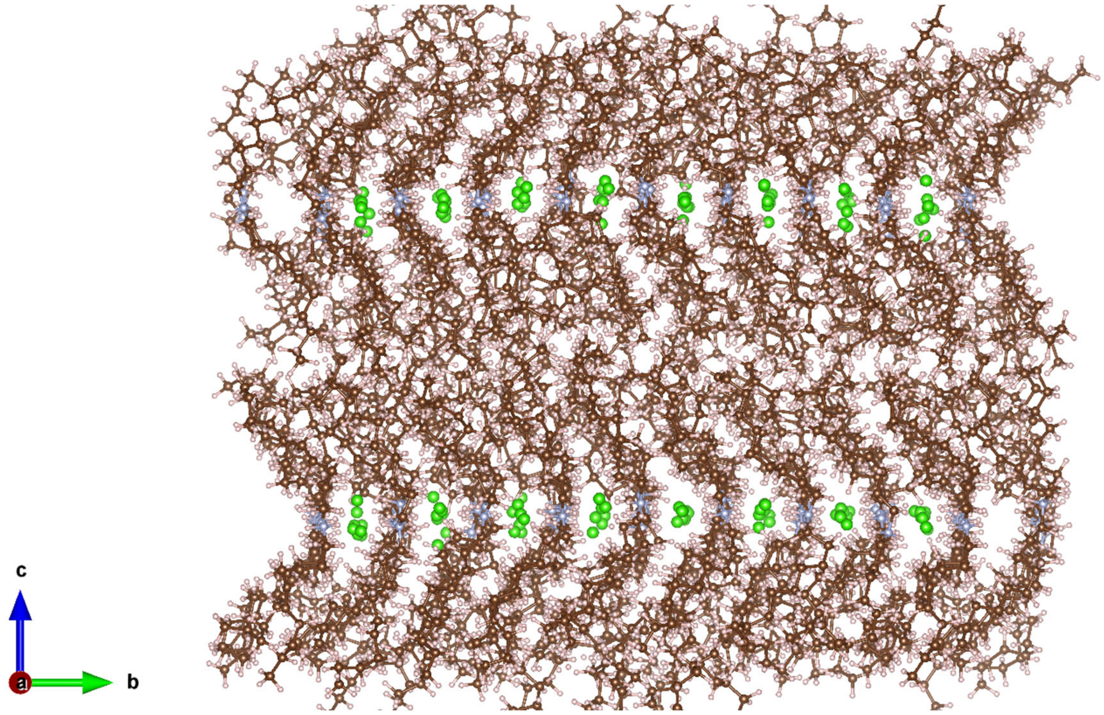

Supplementary Fig. S31 The crystalline structure of $dC_{10}Cl$ at 500 K evolved from the MD simulation.

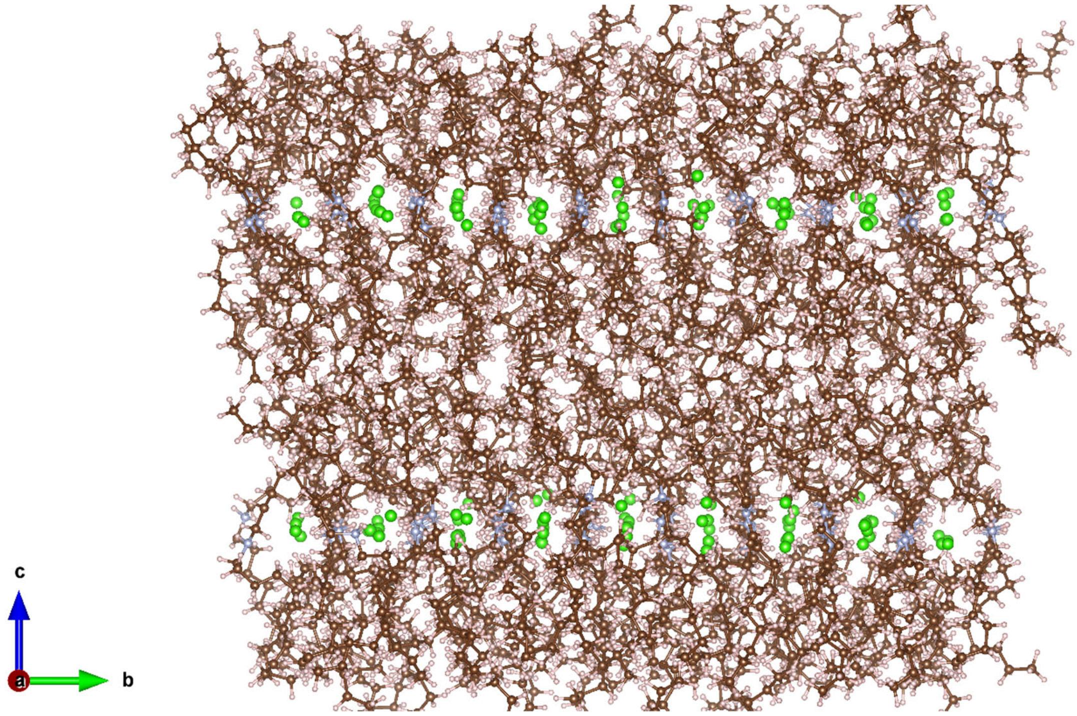

Supplementary Fig. S32 The crystalline structure of $dC_{10}Cl$ at 600 K evolved from the MD simulation.

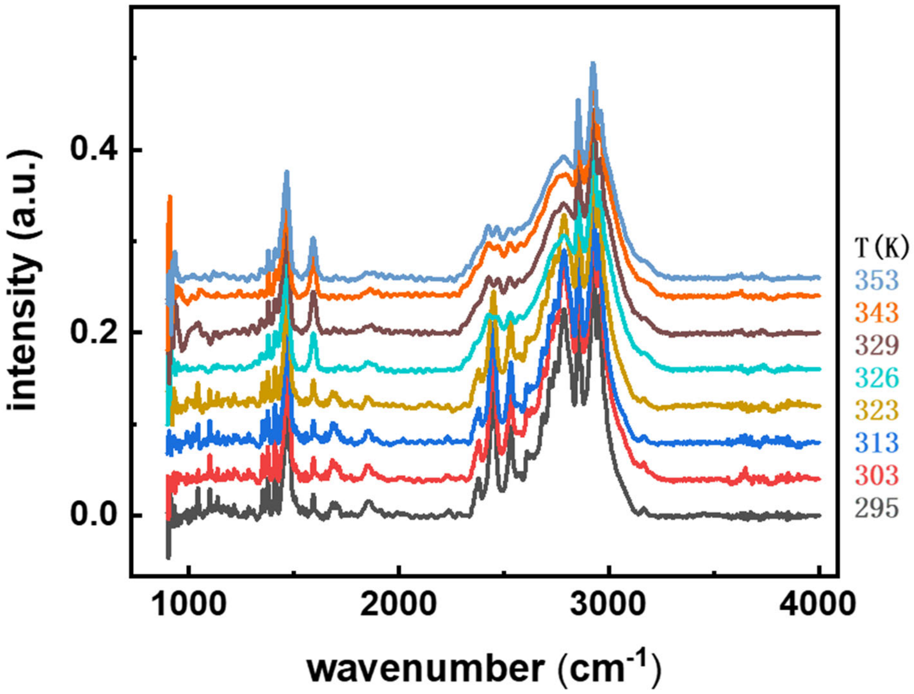

Supplementary Fig. S33 The temperature-variable infrared spectra of $dC_{10}Cl$.

Supplementary Table S1. The thermal properties of phase transition in dC_nX materials. The phase transition temperature on heating (T_s), entropy change under ambient pressure (ΔS), thermal hysteresis and pressure sensitivity of T_s (dT_s/dP) are listed, where sc denotes single crystal while pc denotes polycrystal.

$(C_nH_{2n+1})_2NH_2X$ (dC_nX for short)	T_s (K)	ΔS (J kg^{-1} K^{-1})	hysteresis (K)	dT_s/dP (K $kbar^{-1}$)
dC_6Br (pc)	295	290	10	18.8
dC_8Cl (pc)	298	347	9.3	18.5
$dC_{10}Br$ (sc)	331	364	7	14.3
$dC_{10}Cl$ (pc)	325	400	8	19
$dC_{10}Cl$ (sc)	324	400	8	18.7

Supplementary Table S2. Sample and crystal data for $dC_{10}Cl$ from the SC-XRD at 300 K.

**Chemical formula**	$C_{20}H_{44}ClN$
**Formula weight**	334.01 g/mol
**Temperature**	300 K
**Wavelength**	0.71073 Å
**Crystal size**	0.060 x 0.200 x 0.300 mm
**Crystal system**	Triclinic
**Space group**	P -1
**Unit cell dimensions**	a = 4.9044(13) Å α = 89.630(10) °<br>b = 5.3299(15) Å β = 89.701(10) °<br>c = 43.162(13) Å γ = 88.014(9) °
**Volume**	1127.5(5) $Å^3$
**Z**	2
**Density (calculated)**	0.984 g/cm^3
**Absorption coefficient**	0.169 mm^{-1}
**F(000)**	376

Supplementary Table S3. Data collection and structure refinement for $dC_{10}Cl$ at 300 K for SC-XRD.

**Theta range for data collection**	2.36 to 28.84°
**Index ranges**	-6<=h<=6, -7<=k<=7, -58<=l<=58
**Reflections collected**	23693
**Independent reflections**	5671 [R(int) = 0.0751]
**Coverage of independent reflections**	96.0%
**Absorption correction**	Multi-Scan
**Max. and min. transmission**	0.9900 and 0.9510
**Structure solution technique**	direct methods
**Structure solution program**	SHELXT 2014/5 (Sheldrick, 2014)
**Refinement method**	Full-matrix least-squares on F^2
**Refinement program**	SHELXL-2018/3 (Sheldrick, 2018)
**Function minimized**	Σ w(F_o^2 - F_c^2)^2
**Data / restraints / parameters**	5671 / 2 / 207
**Goodness-of-fit on F^2**	1.070
**Δ/σ_{max}**	0.001
**Final R indices**	4214 data; I>2σ(I) R1 = 0.0728, wR2 = 0.1780<br>all data R1 = 0.0959, wR2 = 0.1913
**Weighting scheme**	w=1/[σ^2(F_o^2)+(0.0663P)^2+0.3785P]<br>where P=(F_o^2+2F_c^2)/3
**Largest diff. peak and hole**	0.267 and -0.334 eÅ^{-3}
**R.M.S. deviation from mean**	0.042 eÅ^{-3}

Supplementary Table S4. Atomic coordinates and equivalent isotropic atomic displacement parameters (Å^2) for dC_{10}Cl at 300 K by SC-XRD. For the atomic positions, see Supplementary Fig. S4.

	x/a	y/b	z/c	U(eq)
Cl1	0.10503(12)	0.32053(9)	0.75001(2)	0.0632(2)
N1	0.7731(3)	0.8273(3)	0.75005(4)	0.0400(4)
C1	0.6069(4)	0.8316(4)	0.72142(5)	0.0464(5)
C2	0.7838(5)	0.8138(5)	0.69305(5)	0.0548(5)
C3	0.6132(5)	0.8171(4)	0.66354(5)	0.0572(6)
C4	0.4905(5)	0.0712(4)	0.65459(5)	0.0552(5)
C5	0.3262(5)	0.0632(4)	0.62505(6)	0.0590(6)
C6	0.2132(5)	0.3136(4)	0.61381(6)	0.0607(6)
C7	0.0493(6)	0.2975(5)	0.58427(6)	0.0643(6)
C8	0.9446(6)	0.5450(5)	0.57156(6)	0.0651(6)
C9	0.7832(7)	0.5264(6)	0.54224(7)	0.0839(9)
C10	0.6857(9)	0.7743(6)	0.52876(8)	0.1036(12)
C11	0.6065(4)	0.8319(4)	0.77864(5)	0.0463(5)
C12	0.7843(5)	0.8135(5)	0.80700(5)	0.0548(5)
C13	0.6146(5)	0.8173(4)	0.83639(5)	0.0568(6)
C14	0.4901(5)	0.0708(4)	0.84549(5)	0.0549(5)
C15	0.3271(5)	0.0631(4)	0.87497(6)	0.0591(6)
C16	0.2128(5)	0.3135(4)	0.88623(6)	0.0603(6)
C17	0.0502(6)	0.2969(5)	0.91568(6)	0.0634(6)
C18	0.9443(6)	0.5453(5)	0.92850(6)	0.0646(6)
C19	0.7841(8)	0.5261(6)	0.95783(7)	0.0840(9)
C20	0.6849(9)	0.7748(6)	0.97112(8)	0.1034(12)

Supplementary Table S5. Selected bond lengths and C–C–C–C dihedral angles of dC_{10}Cl at 300 K from the SC-XRD refinement result. For the atomic positions, see Supplementary Fig. S4.

C–C	1.5 Å	C–N	1.48 Å
C–H	0.97 Å	N–H	0.88 Å
N–H1…Cl1	3.11 Å	N–H2…Cl2	3.14 Å
C1–C2–C3–C4	75.1 °	C2–C3–C4–C5	179.5 °
C3–C4–C5–C6	176.3 °	C4–C5–C6–C7	179.8 °
C5–C6–C7–C8	177.2 °	C6–C7–C8–C9	179.8 °
C7–C8–C9–C10	178.0 °		

Supplementary Table S6. Comparison of the carbon chain refinement result of SC-XRD and PXRD, specifically the carbon bond lengths and C–C–C–C dihedral angles.

bond/angle	SC-XRD	PXRD
C–C	1.5 Å	1.5 Å
C1–C2–C3–C4	75.1 °	75.2 °
C2–C3–C4–C5	179.5 °	179.5 °
C3–C4–C5–C6	176.3 °	176.3 °
C4–C5–C6–C7	179.8 °	179.8 °
C5–C6–C7–C8	177.2 °	177.2 °
C6–C7–C8–C9	179.8 °	179.8 °
C7–C8–C9–C10	178.0 °	178.0 °

Supplementary Table S7. The enthalpy change and entropy change during the phase transition in organic chain-based materials, where $C_{10}H_{22}$[17] undergoing solid-liquid phase transition, while $(C_{10}H_{21})_2NH_2Cl$ and $(C_{10}H_{21}NH_3)_2MnCl_4$[12,13,18] undergoing solid-solid phase transition.

material	enthalpy change (kJ mol^{-1})	entropy change (J K^{-1} mol^{-1})
$(C_{10}H_{21})_2NH_2Cl$ (this work)	43	134
$C_{10}H_{22}$ (*n*-decane)	29	118
$(C_{10}H_{21}NH_3)_2MnCl_4$ (hybrid perovskite)	37	118

Supplementary Note 1. Reduced density gradient (RDG) analysis for clarifying the interaction in $(C_{10}H_{21})_2NH_2Cl$.

To demonstrate the anisotropic interaction in $dC_{10}Cl$ more strongly, we calculated the reduced density gradient (RDG) related to the electron density, which can reveal the intensity of molecular interaction. The calculation results based on the density functional theory (DFT) and the Multiwfn program[19] are shown in Supplementary Fig. S34-S40.

Considering the complexity arising from the multiple-atom-system, the interlayer and intralayer interaction were researched based on 1.5*1.5*1 double-layer and 2*2*0.5 single-layer molecular models, respectively. For two-layer model, RDG of $dC_{10}Cl$ is shown in Supplementary Fig. S34. The downward spike around sign(λ_2)$\rho \sim -0.005$ indicates the weak vdW attractive force, which can be further depicted in the real space with the specific coloring rule in Supplementary Fig. S35. As shown in Supplementary Fig. S35, the vdW interaction of $\rho \sim 0$ and $\lambda_2 \sim 0$ would be depicted in green, and the stronger attractive force can be depicted in color closer to blue. Thus, the vdW interaction can be depicted in Supplementary Fig. S36, and the framed part exhibits the soft interlayer vdW force, which is weaker than the vdW interaction of interchains of each layer.

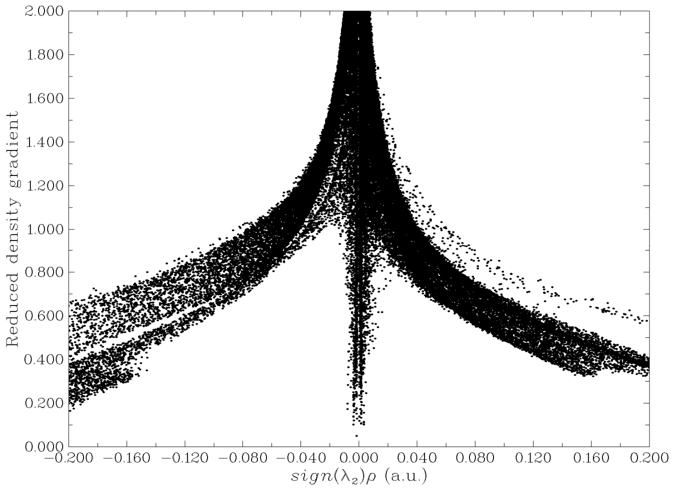

Supplementary Fig. S34 RDG of $dC_{10}Cl$ based on the two-layer molecular model.

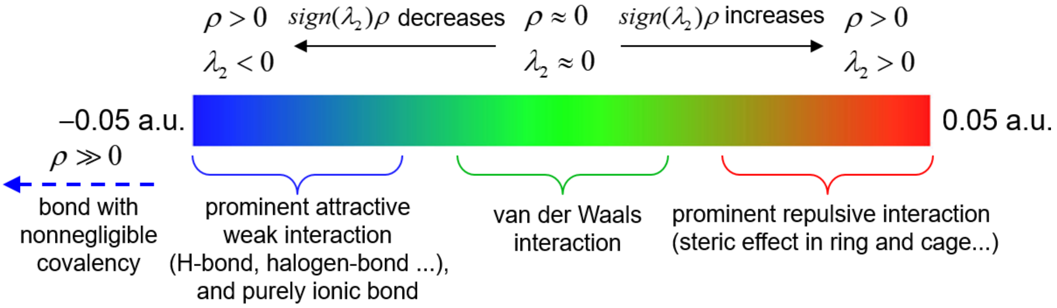

Supplementary Fig. S35 Corresponding colors of the interaction species.

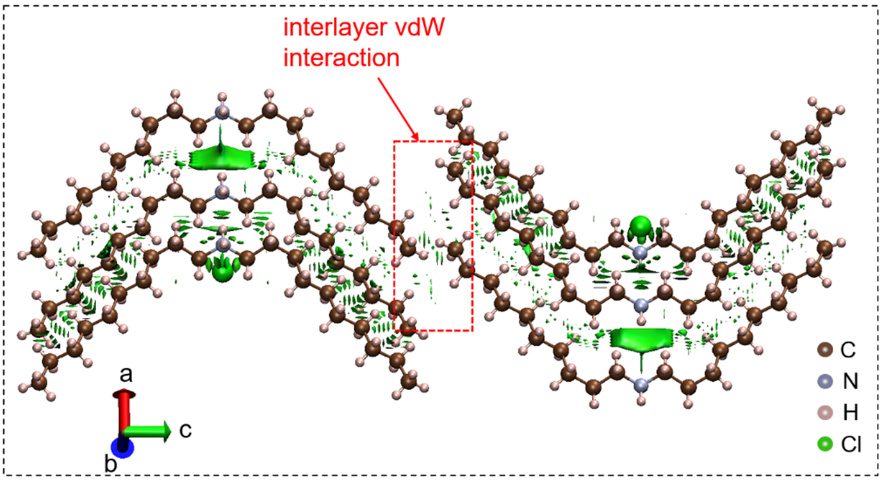

Supplementary Fig. S36 The vdW force in two-layer molecular model.

Moreover, based on the single-layer molecular model, the intralayer interaction can be well illustrated. As shown in Supplementary Fig. S37, the downward spike around sign(λ_2)ρ ~ -0.005 still exists. It refers to the intralayer interchain vdW force, which is also able to be seen in Fig. R3. Located closely left of the vdW spike, the downward spike at sign(λ2)ρ ~ -0.02 indicates the relatively strong hydrogen bond interaction in $dC_{10}Cl$ system, which is further depicted with cyan

electron cloud between N atoms and Cl atoms in Supplementary Fig. S38. Moreover, the downward spike in the range of sign(λ2)ρ ~ -0.24 can indicate the ionic attractive force, as shown in Supplementary Fig. S39 and Supplementary Fig. S40, where the blue electron cloud around the Cl atoms reveals the strong intralayer ionic interaction. It should be noted that the ionic interaction is present along both the *a* and *b* axis in *ab* plane, and ensures the close arrangement of ions in intralayer *ab* plane.

Therefore, combining the RDG results of two-layer and single-layer model, the anisotropic interaction in $dC_{10}Cl$ can be well established, where interlayer weak vdW force, and intralayer strong ionic and hydrogen bond interaction contribute to the 2D layered structure in $dC_{10}Cl$.

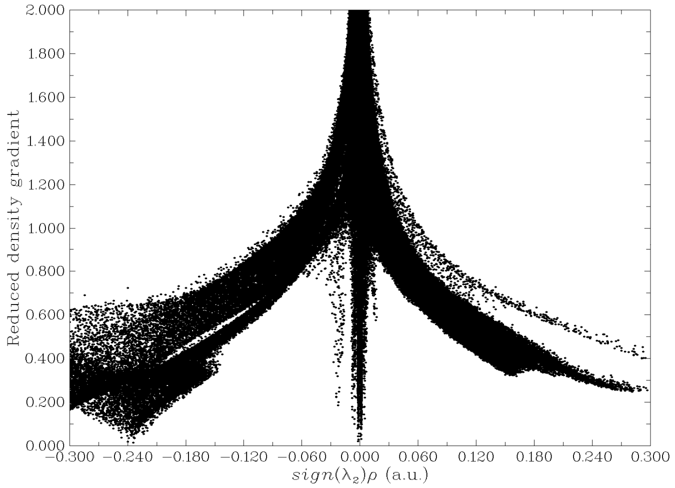

Supplementary Fig. S37 RDG of $dC_{10}Cl$ based on the single-layer molecular model.

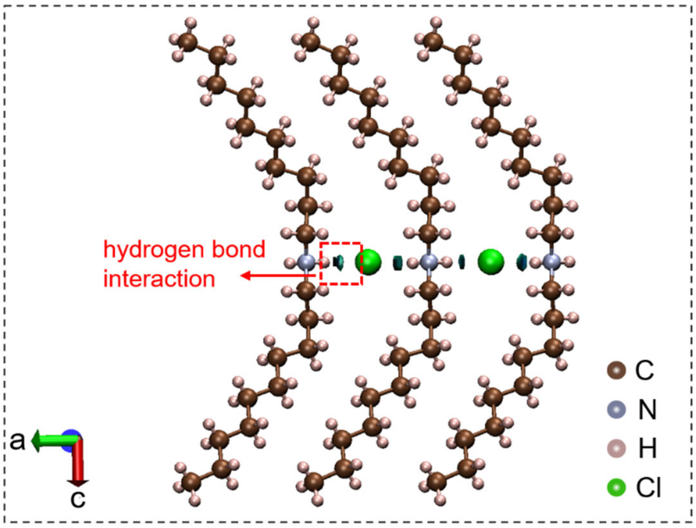

Supplementary Fig. S38 The hydrogen bond interaction in single-layer molecular model.

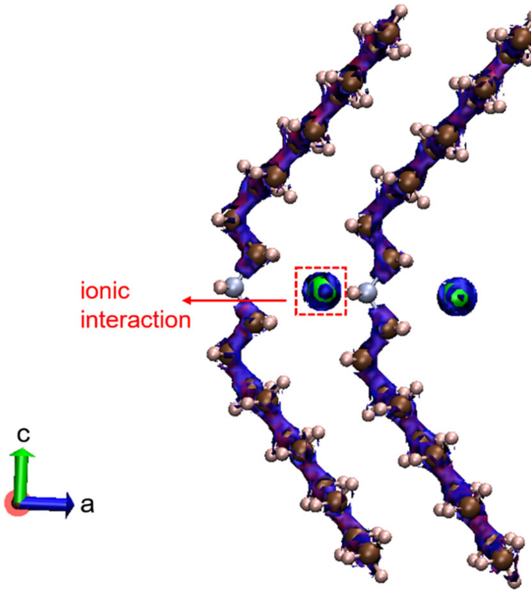

Supplementary Fig. S39 The ionic interaction perpendicular to the *ac* plane in single-layer molecular model.

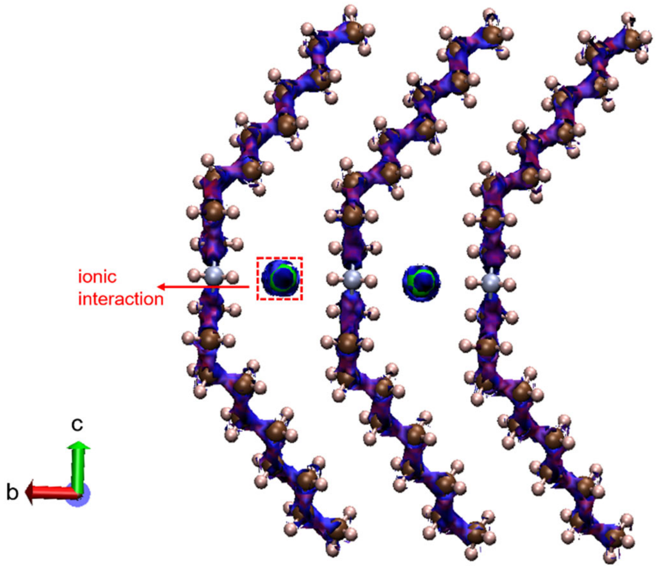

Supplementary Fig. S40 The ionic interaction perpendicular to the *bc* plane in single-layer molecular model.

Supplementary Note 2. Temperature scan rate-dependent hysteresis and consequent barocaloric effect for $(C_{10}H_{21})_2NH_2Cl$.

For exploring the temperature scan rate-dependency of the hysteresis in $dC_{10}Cl$ compound, we performed the DSC measurements at temperature rates of 0.1, 1, 2 K/min, and the appeared hysteresis is 5.9 K, 8 K, 10 K, respectively (Supplementary Fig. S41), while the phase transition entropy change nearly remains constant (~400 J kg^{-1} K^{-1} shown in Supplementary Fig. S42). As a result, the pressure driving the maximum reversible entropy change (~400 J kg^{-1} K^{-1}) slightly enhances to 0.1 GPa at temperature rate 2 K/min, noting this value is 0.08 GPa in the case of 1 K/min.

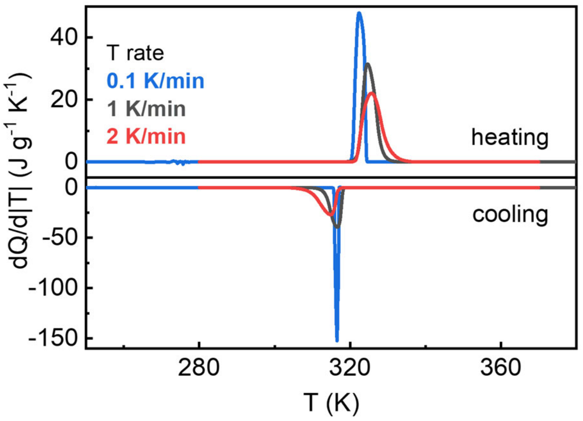

Supplementary Fig. S41 Heat flow of $dC_{10}Cl$ at variable temperature rate of 0.1, 1 and 2 K/min.

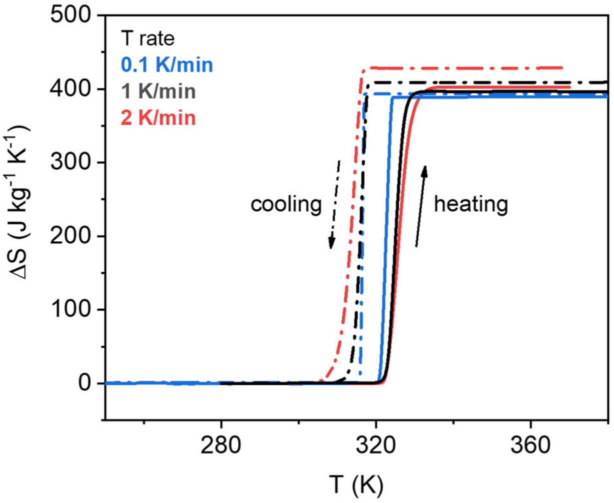

Supplementary Fig. S42 Entropy change of $dC_{10}Cl$ based on the heat flow curves at variable temperature rates.

Supplementary Note 3. Pressure-sensitivity of phase transition from Clausius-Clapeyron equation and its comparison with measured counterpart for $(C_{10}H_{21})_2NH_2Cl$ ($dC_{10}Cl$).

Supplementary Table S8 summaries the dT_s/dP (pressure sensitivity of phase transition temperature) experimentally measured by P-DSC and the one calculated from Clausius-Clapeyron equation for present $dC_{10}Cl$ and many other materials. One can note the non-negligible deviations between the experimental and calculated dT_s/dP for most materials. The difference ratio can be over 30%, and there seems to be a general rule that the measured dT_s/dP is smaller than the one from C-C equation. The key reason might be the penetration of pressure-transmitting gas molecules into the compound, which makes the real pressure exerting on samples lower than the setting value. Hence, a lower dT_s/dP was experimentally detected.

Moreover, most materials listed in Supplementary Table S8 are newly discovered and synthesized, their full physical properties are not yet known, and the ability penetrating by gas molecules is not the same from one material to another, and may vary considerably, so behaving different deviation of dT_s/dP compared to the one from its C-C equation for different materials. For the present 2D plastic crystals of $dC_{10}Cl$, we found that it exhibits high diffusivity in the plastic crystal state above T_s, and can easily stick under a small pressure, but its gas-penetrating ability is not clear to us, and

the related properties are yet to be studied in depth.

Supplementary Table S8. The comparison of dT_s/dP experimentally measured by P-DSC and the one from Clausius-Clapeyron equation. ΔS and ΔV denote the entropy change and volume change, respectively, across phase transition. dT_s/dP (experimentally) is the experimentally measured by P-DSC. dT_s/dP (C-C equation ΔV/ΔS) is the one calculated by Clausius-Clapeyron relation.

Compounds	|ΔS| ($J\ kg^{-1}K^{-1}$)	dT_s/dP ($K\ GPa^{-1}$) (experimentally)	ΔV (E-5 $m^3\ kg^{-1}$)	dT_s/dP ($K\ GPa^{-1}$) (C-C equation ΔV/ΔS)	Error(+-%)	Ref.
$dC_{10}Cl$	400	190	11.9	298	36.2	--
NPG	384	133	4.6	120	10.8	[20, 21]
PG	485	79	5.1	105	24.8	[14, 22]
TRIS	682	37	3.7	54	31.5	[14, 23]
AMP	632	64	4.6	73	12.3	[14, 22]
1-Cl-ada	132	270	4.7	356	24.2	[11]
1-Br-ada	102	333	4	392	15.1	[11]
$Fe_3(bntrz)_6(tcnset)_6$	80	250	2.1	263	4.9	[10]

Supplementary Note 4. Additional barocaloric effect from the volume change outside the phase transition for $(C_{10}H_{21})_2NH_2Cl$ ($dC_{10}Cl$).

Based on the temperature-volume curve shown in Fig. 5c in the main text, we can obtain the change rate of unit cell volume with temperature (dV/dT) ~0.5 E-30 $m^3\ K^{-1}$ at both low-temperature-state and high-temperature-state under atmospheric pressure. Assuming the independent dV/dT on pressure, the additional barocaloric effect ΔS^+ can be obtained with the Maxwell relation of dV/dT = - dS/dP. Therefore, with relation $[\Delta S^+(P) = -(dV/dT)_{p=0}*P]$, additional entropy change can be estimated as -9 $J\ kg^{-1}\ K^{-1}$ under pressurization of 20 MPa and -45 $J\ kg^{-1}\ K^{-1}$ under pressurization of 100 MPa, which emphasizes that the volume expansion in each phase contributes to the enhanced barocaloric effect.

Supplementary References

1. Yuce, S. *et al.* Barocaloric effect in the magnetocaloric prototype $Gd_5Si_2Ge_2$. *Appl. Phys. Lett.* **101**, 071906 (2012).
2. Bocca, J. R. *et al.* Giant barocaloric effect in commercial polyurethane. *Polymer Testing* **100**, 107251 (2021).
3. Carvalho, A. M. G., Imamura, W., Usuda, E. O. & Bom, N. M. Giant room-temperature barocaloric effects in PDMS rubber at low pressures. *Eur. Polym. J.* **99**, 212–221 (2018).
4. Usuda, E. O., Bom, N. M. & Carvalho, A. M. G. Large barocaloric effects at low pressures in natural rubber. *Eur. Polym. J.* **92**, 287–293 (2017).
5. Matsunami, D., Fujita, A., Takenaka, K. & Kano, M. Giant barocaloric effect enhanced by the frustration of the antiferromagnetic phase in Mn_3GaN. *Nat. Mater.* **14**, 73–78 (2015).
6. Liu, Y. *et al.* Large barocaloric effect in intermetallic $La_{1.2}Ce_{0.8}Fe_{11}Si_2H_{1.86}$ materials driven by low pressure. *NPG Asia Mater.* **14**, 30 (2022).

7. Mañosa, L. *et al.* Inverse barocaloric effect in the giant magnetocaloric La–Fe–Si–Co compound. *Nat. Commun.* **2**, 595 (2011).
8. Strässle, Th., Furrer, A., Hossain, Z. & Geibel, Ch. Magnetic cooling by the application of external pressure in rare-earth compounds. *Phys. Rev. B* **67**, 054407 (2003).
9. Zhang, Z. *et al.* Thermal batteries based on inverse barocaloric effects. *Sci. Adv.* **9**, eadd0374 (2023).
10. Romanini, M. *et al.* Giant and reversible barocaloric effect in trinuclear spin‐crossover complex $Fe_3(bntrz)_6(tcnset)_6$. *Adv. Mater.* **33**, 2008076 (2021).
11. Aznar, A. *et al.* Reversible colossal barocaloric effects near room temperature in 1-X-adamantane (X=Cl, Br) plastic crystals. *Applied Materials Today* **23**, 101023 (2021).
12. Gao, Y. *et al.* Reversible colossal barocaloric effect dominated by disordering of organic chains in $(CH_3–(CH_2)_{n-1}–NH_3)_2MnCl_4$ single crystals. *NPG Asia Mater.* **14**, 34 (2022).
13. Li, J. *et al.* Colossal reversible barocaloric effects in layered hybrid perovskite $(C_{10}H_{21}NH_3)_2MnCl_4$ under low pressure near room temperature. *Adv. Funct. Mater.* **31**, 2105154 (2021).
14. Aznar, A. *et al.* Reversible and irreversible colossal barocaloric effects in plastic crystals. *J. Mater. Chem. A* **8**, 639–647 (2020).
15. Salvatori, A. *et al.* Colossal barocaloric effects in adamantane derivatives for thermal management. *APL Materials* **10**, 111117 (2022).
16. Seo, J., Braun, J. D., Dev, V. M. & Mason, J. A. Driving Barocaloric Effects in a Molecular Spin-Crossover Complex at Low Pressures. *J. Am. Chem. Soc.* **144**, 6493–6503 (2022).
17. Domalski, E. S. & Hearing, E. D. Heat capacities and entropies of organic compounds in the condensed phase. Volume III. *J. Phys. Chem. Ref. Data* **25**, 1 (1996).
18. Seo, J. *et al.* Colossal barocaloric effects with ultralow hysteresis in two-dimensional metal–halide perovskites. *Nat. Commun.* **13**, 2536 (2022).
19. Lu, T. & Chen, F. Multiwfn: A multifunctional wavefunction analyzer. *J Comput Chem* **33**, 580–592 (2012).
20. Li, B. *et al.* Colossal barocaloric effects in plastic crystals. *Nature* **567**, 506–510 (2019).
21. Lloveras, P. *et al.* Colossal barocaloric effects near room temperature in plastic crystals of neopentylglycol. *Nat. Commun.* **10**, 1803 (2019).
22. Tamarit, J. Li., Legendre, B. & Buisine, J. M. Thermodynamic study of some neopentane derivated by thermobarometric analysis. *Mol. Cryst. Liq. Cryst.* **250**, 347–358 (1994).
23. Eilerman, D. & Rudman, R. Polymorphism of crystalline poly(hydroxymethyl) compounds. III. The structures of crystalline and plastic tris(hydroxymethyl)aminomethane. *The Journal of Chemical Physics* **72**, 5656–5666 (1980).
